# Integrating non-technical skills into undergraduate medical simulation: a scoping review and thematic analysis of current practices

**DOI:** 10.1186/s41077-025-00377-9

**Published:** 2025-10-21

**Authors:** Shen Chuen Khaw, Lexzion Chung, Nigel Fancourt

**Affiliations:** 1 Department of Education, University of Oxford, Oxford, UK; 2 School of Medicine, University of Dundee, Dundee, UK

**Keywords:** Non-technical skills, Undergraduate medical simulation, Patient safety, Medical student, Human factors

## Abstract

**Background:**

Non-technical skills (NTS) play a crucial role in reducing patient harm. In light of this, medical schools are now integrating NTS into their undergraduate curricula to encourage efficiency and reduce human errors, particularly during highly stressful scenarios. Utilising medical simulations has become prevalent for honing these skills in a controlled environment. However, a lack of guidance on how best to design and deliver effective NTS training leads to inconsistencies in quality and outcomes. Therefore, this review aims to examine current practices in simulation-based NTS training for medical students to collate evidence-informed strategies for enhancing its effectiveness.

**Methods:**

A scoping review was carried out according to PRISMA-ScR guidelines. A search strategy was performed on PubMed, Scopus and Web of Science databases from inception to 17th June 2025. A thematic analysis of eligible studies identified recurring themes, leading to a conceptual model for the current delivery of NTS training.

**Results:**

The screening process yielded 51 articles and commonly occurring themes were synthesised: Simulation setup, simulation modality, post-simulation activity, observational tools for NTS and learning environment. We identified several key practice points that are essential for the successful implementation of NTS training. These include pre-simulation briefings, appropriate fidelity, and debriefing sessions which collectively form the foundation for effective training outcomes.

**Conclusion:**

The resulting themes highlight the effective strategies currently employed for NTS training in undergraduate medical simulation. Educators will be able to use these to design and implement consistent, effective NTS training.

**Supplementary Information:**

The online version contains supplementary material available at 10.1186/s41077-025-00377-9.

## Introduction

Non-technical skills (NTS) such as communication, leadership, teamworking, interprofessional collaboration, decision-making, and prioritisation are increasingly recognised as important elements in reducing patient harm [1]. These skills are particularly crucial in high-pressure clinical environments such as emergency departments and operating theatres, where rapid and coordinated responses are essential to ensure patient safety [2–4].

In response, undergraduate medical education has increasingly recognised the importance of NTS, incorporating them into curricula with the aim of improving clinical performance and reducing preventable errors [5]. Simulation-based training has emerged as a key modality for this purpose, offering a safe, controlled and immersive environment where learners can develop and apply both technical and non-technical competencies [2, 6]. Despite widespread adoption and a growing body of literature supporting the effectiveness of NTS interventions in improving safety outcomes [3, 7–9], there remains a lack of consensus regarding the most effective pedagogical approaches for facilitating high-quality learning experiences and the optimal methods for developing NTS [10–12]. The variability in practices leads to uncertainty regarding the effective structure and delivery of NTS training through simulation, leading to inconsistencies in educational quality and learner outcomes across different medical programmes.

To address this gap, this article will examine how NTS training is currently being implemented in undergraduate medical simulations. We anticipate that our findings will highlight evidence-based NTS training practices that can be adopted to ensure consistent, high-quality NTS education in undergraduate medical curricula.

## Materials and methods

In this review, the guidelines outlined in the Preferred Reporting Items for Systematic Reviews and Meta-Analyses extension for Scoping Reviews (PRISMA-ScR) were closely followed [13]. This study protocol is registered in OSF Registries OSF.IO/NF96W [14]. The goals of scoping reviews are diverse, including the summarisation and dissemination of current research findings, the evaluation of whether a more extensive systematic review is viable and the identification of gaps in our current understanding within the existing literature [14]. To guide the scoping review process, we adopted the established five-stage framework developed by Arksey and O’Malley (2005), which provides a systematic approach to synthesising and mapping existing literature [15]. This framework is widely utilised in scoping reviews to ensure comprehensive exploration and organisation of data, particularly in fields with diverse and heterogeneous research landscapes [16–18]. Below, we outline each stage of this framework as applied to our study.

### Stage 1: identifying the research question

Our study aims to map the existing literature on simulation-based NTS training in undergraduate medical education, identify common practices and implementation strategies and highlight gaps to inform future educational approaches.

### Stage 2: identifying relevant studies

A comprehensive literature search was conducted on PubMed, Scopus and Web of Science databases using the specified keywords, namely ‘non-technical skills’, ‘human factors’, ‘human ergonomics’, ‘human behaviour’, ‘behavioural skills’, ‘medical simulation’, ‘clinical simulation’, ‘surgical simulation’, ‘undergraduate medical education’, ‘medical student’, ‘undergraduate doctor’. The search strategy for individual databases is detailed in Appendix 1. Articles were limited to English from inception up to 17th June 2025. No limits were set on study design. The inclusion and exclusion criteria are listed in Appendix 2.

### Stage 3: study selection

**Fig. 1:**
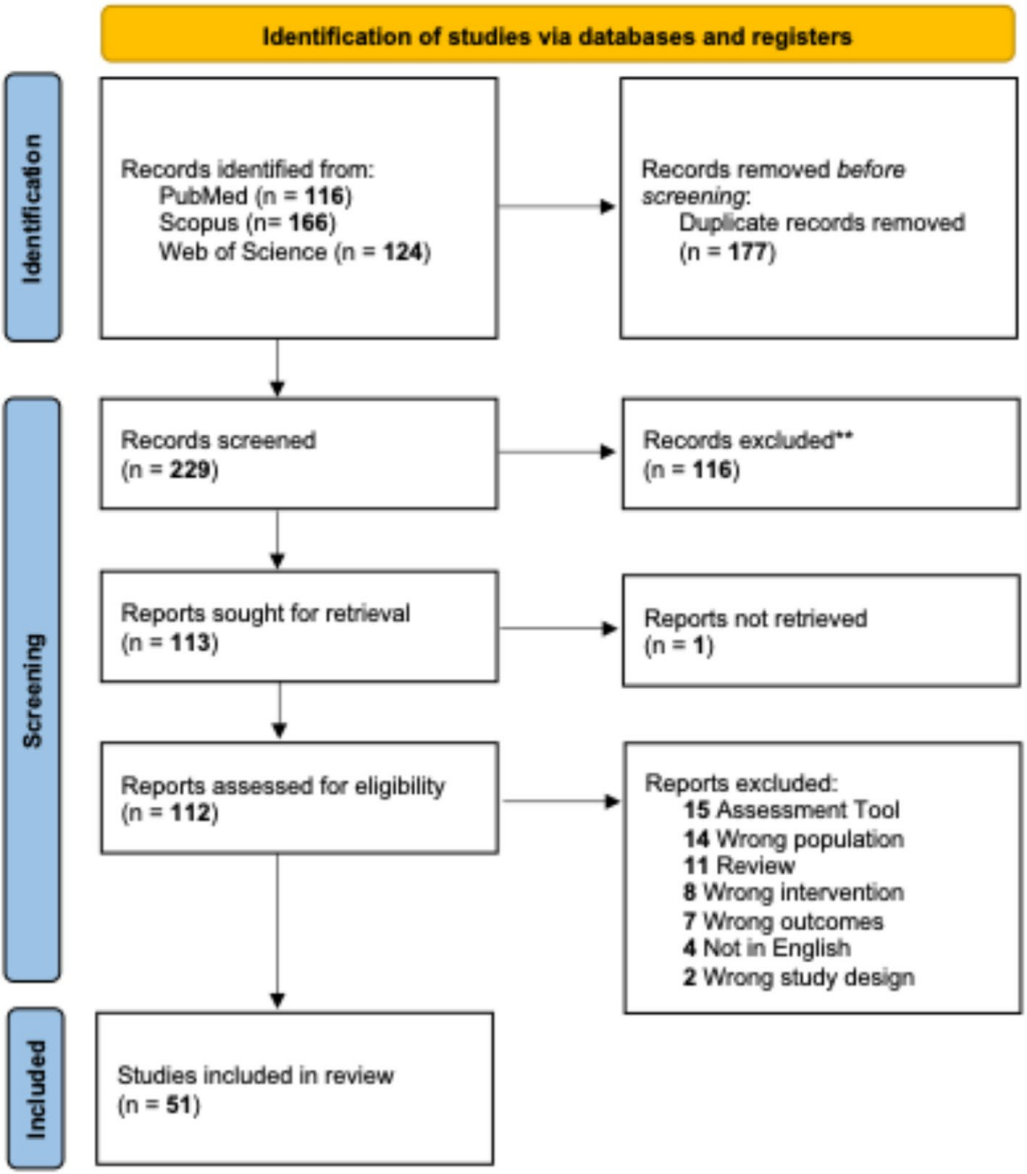
PRISMA flowchart [23]

The Covidence Systematic Review Software was applied to streamline the identification and removal of duplicates and facilitate article selection [19]. The manual screening process was carried out by two members of the review team independently (SCK and LC) who meticulously assessed the identified records. The reference lists of included articles were screened to identify any additional relevant studies that may have been missed during the initial database search. Full texts of relevant articles were retrieved and individually reviewed by both reviewers for final inclusion, with any conflicts resolved by consensus. Upon encountering one abstract where the full-text article was unavailable, we contacted the authors for clarification, and they confirmed that a full-text version of the article had not been published. The search process is detailed in Fig. 1.

#### Quality assessment

The quality of the included articles was assessed using the Joanna Briggs Institute (JBI) critical appraisal checklists [20–22].

### Stage 4: charting the data

A standardised form was pilot tested and utilised to extract various data fields, encompassing study registration details (author name, publication year, country and ethical approval), study design components (population characteristics, simulation type, data collection methods and interventions) and specifics regarding the delivery of NTS (including the particular NTS conveyed, and the methods employed for their assessment). Ethics approval was deemed unnecessary as the focus of this study was directed towards the analysis of previously published scientific articles; however, we did verify that the underlying articles themselves were conducted ethically.

### Stage 5: collating, summarising and reporting the results

The thematic analysis was conducted according to Braun and Clarke’s framework to collate, summarise, and interpret the findings [24]. This involved a systematic process of familiarising with the extracted data through repeated reading and initial notetaking, followed by inductive coding to identify meaningful features. The codes were then reviewed and organised into potential themes, ensuring both semantic and latent content were captured. Themes were refined and defined to provide a coherent narrative that synthesized the data, highlighting key patterns and insights relevant to the research question.

#### Reflexivity

As the authors of this review, we acknowledge the presence of our personal perspectives and potential biases that might impact how we interpret and present the reviewed literature. Given backgrounds in medical education, its research and our own experiences, we recognise that these factors could influence our comprehension of the articles. In mitigating potential biases, a deliberate endeavour has been undertaken to engage with the literature objectively, in Bird’s sense [25], diligently scrutinising the methodologies, findings and implications of each study. It is crucial for readers to understand that these subjectivities are woven into any textual review. Indeed, more strongly, we would follow Gadamer’s claim that we are hermeneutically informed by our pre-understandings and could not come to any interpretive discernment without them [26]. Hence, the consideration of different perspectives and exploration of additional research is highly encouraged to obtain a more comprehensive understanding of the topics being discussed.

## Results

A comprehensive search of the databases yielded 406 articles spanning from inception to 17th June 2025. Following the elimination of 177 duplicates, the remaining 229 articles underwent title and abstract screening. 113 articles met the criteria for full-text screening based on inclusion and exclusion parameters. After a meticulous review, 62 articles were excluded with reasons outlined. The final inclusion consisted of 51 articles. A visual representation of the screening process is presented in the PRISMA Flowchart (Fig. 1). Most of the included studies were found to be of high quality [5, 27–73], while three studies [74–76] were rated as fair quality for reasons listed in Appendix 3. The diverse current practices, as reported by various studies, are detailed in Supplementary Tables 1–5. These tables encompass pre-simulation activities, simulation activities, post-simulation debriefing and feedback, the implementation of NTS and the assessment tools employed.

A total of 47 studies examined teamwork, followed by 26 studies on situational awareness, 25 studies each on leadership and prioritisation, 24 on decision making, 10 on safety behaviours, 9 on patient communication and 6 studies addressed wellbeing.

The included studies were conducted across a range of countries, reflecting a diverse international representation. Most studies were conducted in the UK (*n* = 15), followed by Germany (*n* = 9), and other countries including Portugal (*n* = 2), Australia (*n* = 2), Norway (*n* = 2), Italy (*n* = 2), the USA (*n* = 2), Canada and Brazil (joint study, *n* = 1), France (*n* = 1), Finland (*n* = 1), South Korea (*n* = 1), Poland (*n* = 1), Greece (*n* = 1), Switzerland (*n* = 1), Hong Kong (*n* = 1), Dominican Republic (*n* = 1) and Morocco (*n* = 1). Several studies were also conducted at a multi-national level, involving countries such as Germany, Italy, Belgium, Netherlands, Romania, Portugal, Syria and broader European regions. The details are outline in Supplementary Table 4.

### Theme 1: simulation setup

#### Subtheme 1: technical knowledge

Pre-simulation lectures on technical knowledge were perceived as beneficial, as they facilitated foundational understanding and skill acquisition, improving readiness for simulation [50, 58]. A solid technical knowledge base may be a prerequisite to high-fidelity simulations for maximal educational impact [33]. While post-session lectures were deemed less beneficial by students, debriefings were particularly effective when they emphasised complex issues over basic clinical management skills [37, 58]. Thus, both technical and non-technical skills enable students to provide effective patient centred care [66].

#### Subtheme 2: spotlighting non-technical skills

Pre-simulation NTS training has shown positive effects on student performance by laying a foundation for learning during the simulation [5, 28, 39, 57, 75]. The simulation sessions that followed training enabled students to contextualise and apply their learning into practise [34, 38, 69], enhancing their confidence and skill acquisition [28, 49, 50, 75]. Video demonstrations and structured training programs helped students visualise expectations, guided their behaviour and serve as valuable memory aids [31, 33, 37, 46, 57]. These structured strategies in simulation activities highlighted the importance of NTS, providing opportunities to practice and improve them [42, 48, 52].

#### Subtheme 3: repetition

Planned repetition and vertical integration of simulation and NTS training significantly improved performance [5, 38, 39]. Research indicated that multiple simulation sessions led to higher NTS scores [47, 62]. Sequential simulation exercises encouraged students to apply reflections from previous sessions to new scenarios, promoting continuous learning and improvement [31, 46, 68].

#### Subtheme 4: resource considerations

Simulation-based training is resource-intensive, requiring significant financial investment due to the need for skilled personnel and specialised equipment [5, 43, 66]. VR simulations additionally required specific headsets, which increased costs [58]. Cost-saving strategies included using students as standardised patients (SP) and employing lower-fidelity manikins, which reduced the financial burden without compromising the educational experience [52].

### Theme 2: simulation modality

#### Subtheme 1: simulation activity

Group-based simulation exercises with one student as a team leader led to higher NTS scores compared to simulations involving solo student practice [53]. Group simulations also encouraged peer learning through experience sharing, and role relevance motivated learning [27, 61]. Other simulation modalities, such as VR and immersive ward-round scenarios, provided realistic contexts for student practice [58, 69, 71]. Additionally, allowing students to take the role of the patient enhanced empathy and communication skills [40, 69]. These activities can be tailored to suit the context and learning objectives of the simulation session.

#### Subtheme 2: fidelity

High-fidelity simulations, while effective in building confidence and offering realistic practice environments, posed challenges due to increased stress levels and financial costs [33, 56, 67]. Some studies indicated that performance might be better in lower-fidelity scenarios, as higher stress could impact skill application [55]. Despite this, high fidelity remained a preferred option among students for its immersive quality and teamwork facilitation [48, 55].

#### Subtheme 3: interprofessional education

Simulation with interprofessional education (IPE) elements enhanced teamwork, communication, situational awareness and role understanding [35, 60, 68]. Through these experiences, students learned to appreciate the roles and responsibilities of various health professionals, fostering respect and collaboration [54, 68, 70].

### Theme 3: post-simulation activities

#### Subtheme 1: reflection

Post-simulation reflection allowed students to objectively assess their actions, and they were more open to change when insights emerged from their own discoveries [43, 45, 63]. Sequential simulation supported experiential learning by encouraging students to apply reflections in subsequent scenarios [68]. Reflection activities highlighted improvement areas, which motivated students to advance their skills [31, 44]. Some sessions included ‘pause and reflect’ moments, allowing students to stop and discuss their actions, fostering critical thinking and planning [46].

#### Subtheme 2: debriefing

Debriefing was essential for skill acquisition, with self- or peer-led debriefing proving useful in driving personal responsibility in learning [29, 41, 45, 63]. Structured frameworks for debriefing facilitated comprehensive discussions, especially useful for novice participants, though more experienced learners may benefit from less structured formats [72, 74]. Video-assisted debriefing provided additional review support and encouraged reflection [62].

#### Subtheme 3: feedback

High-quality, individualised feedback was essential for skill improvement and was valued highly by students [52, 63, 69]. Expert feedback improved performance, and bite-sized or written feedback was particularly beneficial in high-stress environments, as it allowed students to revisit recommendations at their own pace [54, 55].

### Theme 4: observational assessment tool for NTS

#### Subtheme 1: validated tools

Standardised tools for assessment in simulation scenarios facilitated objective self-assessment and reflection, providing students with clear metrics to evaluate their own performance. This allowed for more precise self-assessment, promoting continual improvement and self-awareness [42, 43, 45]. The use of validated assessment tools also enhanced the accuracy and reliability of skill evaluation, offering standardised criteria for NTS and consistent assessment across students [5, 39, 51, 67].

The structure provided by these tools improved the quality and specificity of feedback, as students could more easily identify precise areas for improvement in NTS, which in turn supported targeted skill development [44, 56, 59]. By using consistent, validated measures, feedback could be more structured and beneficial for the students'ongoing learning process.

#### Subtheme 2: adaptation of assessment tools

In some cases, assessment tools were adapted to address logistical and contextual challenges. For example, iTOFT was modified to facilitate evaluation of entire groups, which enabled assessors to gauge collective performance and dynamics effectively in team-based scenarios [56]. However, other studies indicated that certain tools proved difficult to use when assessing groups of students simultaneously, as they were initially designed for individual assessments [59].

To align with specific educational objectives, institutions adapted standardised tools such as NOTECHS, modifying them to better suit their educational context and goals, ensuring assessments remained relevant and practical for their programs [32]. However, some researchers noted that standardised tools might not fully capture nuanced aspects of performance, suggesting that tool flexibility is sometimes necessary for accurate and meaningful evaluation [32].

### Theme 5: learning environment

#### Subtheme 1: psychological safety

A familiar and psychologically safe learning environment was crucial for reducing anxiety and promoting learning [27, 67]. Having familiar faculty as facilitators encouraged students to ask questions and participate actively[48].

#### Subtheme 2: level of challenge

Effective learning was achieved when extrinsic stress levels were controlled. While exposure to challenging situations allowed students to develop coping strategies, overly high-stress levels negatively affected performance and feedback absorption [5, 55, 69]. A supportive, low-pressure environment fostered focus and skill prioritisation, allowing students to internalise relevant techniques [27, 30].

#### Subtheme 3: supportive learning environment

Supportive environments promoted enhanced learning experiences, with students benefiting from a culture where mistakes were acceptable and emotional decompression was encouraged [49, 61]. Approachable, near-peer tutors contributed to a safe and supportive simulation setting, where students felt empowered to ask questions and experiment [43, 73]. A summary of the themes and sub-themes are described in Table 1.

**Table 1:** Themes and sub-themes

Theme	Sub-themes	Explanation
Simulation setup	Technical knowledge	Providing essential technical information prior to simulation enriches the learning experience and redirects the emphasis onto NTS
	Spotlighting NTS	Empowering learners to recognise and apply NTS through dedicated NTS teaching and modelling prior to simulation
	Repetition	Optimising knowledge accumulation through prolonged learning facilitating continuous skill enhancement
	Resource	High-quality simulations necessitate adequate resources, staffing and proficient training for effective implementation and learning
Simulation modality	Simulation activity	VR, game, disaster, ward-based
	Fidelity	High-fidelity (realism) environments can be used to enhance the experiential realism in a simulation
	Interprofessional education	Multiprofessional team participation in simulation enhances communication and collaboration for effective patient care
Post-simulation activity	Reflection	Facilitating reflection and condensing learning through crucial post-simulation sessions
	Debriefing	Identifying best practices and addressing learning needs through structured post-simulation debriefing
	Feedback	Timely feedback aids learning absorption, preventing overload and initial shock
Observational assessment tool of NTS	Validated tools	Utilising validated assessment tools for NTS enhances the overall effectiveness and precision of the learning process
	Adaptation of assessment tools	NTS assessment frameworks should be adaptable to developmental needs
Learning environment	Psychological safety	Ensuring psychological safety from pre-simulation activities through immersion to post-simulation debriefing is vital for cultivating positive learning behaviours among learners
	Level of challenge	Tailoring stress optimally to ensure learners within the zone of proximal development enhances learning and performance
	Supportive learning environment	The catalytic influence of a supportive learning environment on the learning process

## Discussion

Prior to delving into effective implementation strategies for NTS training, it is imperative to establish a shared understanding of the terminology commonly employed in the context of ‘non-technical skills’. Nestel and colleagues [77] have contended that ‘the term NTS is misleading, inaccurate and oversimplifies critical aspects of professional clinical practice’ and have proposed alternative terms such as ‘human factors’.

In contrast, Murphy [78] has suggested using the term ‘behavioural skills’ instead. However, the term ‘non-technical skills (NTS)’ has been employed in our study as it encompasses a broader spectrum of competencies than behavioural skills alone. The term ‘behavioural skills’ may be misleading, as they primarily relate to interpersonal attributes and teamwork, while NTS also encompass essential cognitive and social abilities such as decision-making, situational awareness, and prioritisation, which are critical for safe and effective patient care [7, 79]. Alternative terms such as human factors and ergonomics could carry connotations of individual blame, and ‘ergonomics’ is typically associated with system-level design rather than individual performance. Importantly, within the medical community, the term NTS is widely recognised and valued. Medical professionals generally acknowledge its significance, and key bodies such as the General Medical Council and the UK Royal Colleges have endorsed the term by incorporating NTS into professional development and training programmes [80, 81]. Hence, we feel that the term ‘non-technical skills’ is still currently relevant, aligning with the GMC’s Generic Professional Capabilities Framework who have listed NTS as a key capability for patient safety [80]. Lastly, we find that the work by O’Connor and O’Dea [79] has thoughtfully articulated many of the perspectives we share.

Our findings suggest that pre-simulation preparation for both technical and non-technical skills is foundational to NTS simulation training [5, 33, 39, 50, 58, 75]. Familiarising students with technical knowledge may reduce the cognitive burden of recalling this knowledge, allowing them to focus on learning and practising NTS [82, 83]. Providing structured guidance through teaching and demonstrations would allow students to observe, internalise and emulate exemplary NTS practices [39, 84, 85].

Interestingly, students benefited more from pre-simulation lectures and training compared to post-session lectures, indicating that cognitive readiness prior to simulations optimizes learning outcomes [37, 58]. These findings echo the need for a paradigm shift: simulation should no longer be viewed as an isolated learning event but as part of an integrated sequence where preparation and reflection are equally emphasised [86–88].

The findings around repetition reinforce its role as a critical driver of skill retention and performance enhancement. However, it is not mere repetition but *structured*, *reflective repetition* that yields the best results [43, 45, 63, 68]. The opportunity for students to apply lessons from one scenario to another builds iterative learning loops, encouraging a deeper understanding of underlying principles rather than rote memorisation [89, 90].

High-fidelity simulations, while preferred for their immersive quality, present challenges of stress and resource demands [33, 56, 67]. The observation that high-fidelity simulations may elevate stress levels to the point of diminishing returns highlights the importance of managing cognitive load [55, 69, 91]. The thematic analysis reveals that lower-fidelity options can achieve comparable outcomes for specific learning objectives, particularly when integrated with reflection and feedback [55]. These findings highlight the complexity of factors influencing simulation learning, challenging a straightforward link between fidelity levels and learning outcomes [92]. Therefore, the simulation should be tailored to participants’ training levels, ensuring adequate duration and incorporating engaging scenarios with a thoughtfully calibrated level of stress to optimise effectiveness.

Although simulation may reduce the risk towards real patients, it can still induce psychological distress for learners as they are placed in unfamiliar, high-stakes scenarios [93–95]. Consequently, establishing a psychologically safe environment is essential to support continuous learning and skill development in simulation [96–99]. Psychological safety mitigates performance anxiety while encouraging learners to focus on refining skills, seek constructive feedback and take ownership of learning [6, 96, 98].

Debriefing and feedback remain as indispensable components in simulation, providing learners with an opportunity to engage in thoughtful reflection and responsive analysis of their interpretations of the activity [85].

Several studies have also highlighted the significance of timely debriefing or feedback tailored to the learner’s needs [51, 54, 69, 100]. Debriefing encompasses a comprehensive process that involves addressing emotions and excitement of our learners, while feedback is more specifically targeted towards skill development and improvement. When conducted immediately post-simulation, debriefing leverages the temporal proximity of learning to enhance recall and internalisation [27, 63, 101, 102]. However, some studies have shown that debriefing should instead begin by addressing the students’ experiences and emotions to support reflection rather than retrospective correction alone[102–105]. Therefore, educators should carefully optimise the timing of feedback to maximise educational impact.

Another intriguing finding is that allowing students to assume the role of the patient during simulations fosters a development of empathy and awareness [40, 106]. This could be further enhanced by integrating real patient narratives or feedback into the debriefing process, providing an additional layer of authenticity and insight.

Novel simulation technologies such as virtual reality (VR), immersive ward scenarios and video-assisted debriefings promote engagement, reflection and skill development [58, 62, 71, 107]. Additionally, AI-powered performance analytics offer objective assessments and personalised feedback, supporting adaptable, scalable teaching and learning programs [108–110]. However, Pears (2024) has cautioned that while AI tools can support knowledge acquisition, they often fall short in delivering conceptual depth, accurate factual feedback and effective language use [111]. Additionally, the current lack of ethical guidelines and issues surrounding data privacy have limited the integration of AI in educational settings [109]. As such, it is important that future research is carried out to rigorously evaluate the educational impact of AI-driven simulation learning on a background of comprehensive ethical and regulatory frameworks. Only when we ensure the safety of our learners can we fully unlock the potential of AI in medical simulation.

Crucially, tailoring simulation activities to align with learning objectives enables students to engage with scenarios that directly address their developmental requirements, thereby facilitating more effective skill acquisition and application [27, 29, 53, 61, 75].

Standardised tools, such as NOTECHS and iTOFT, support consistent, reliable and structured feedback especially for novice assessors [2, 4, 112, 113]. However, their rigid frameworks may not fully capture the unique dynamics of specific educational contexts, such as cultural nuances, institutional priorities, or group-based simulations [32, 59, 114–116].

The development of the Medical Students’ Non-Technical Skills (Medi-StuNTS) behavioural marker system was prompted by concerns that existing tools may not adequately reflect the behaviours and developmental needs of medical students in simulation-based acute care settings [117]. This stems from the recognition that medical students, as novices in clinical environments, differ significantly from more experienced trainees in both performance and learning needs [118–121]. The authors have supported the need for tailored assessment tools based on learner experience and setup. Rather than adopting a one-size-fits-all approach, educators and researchers should aim to adapt validated tools to align with the specific educational objectives, learner profiles and situational demands of each simulation setting [114–116].

It is noteworthy that while the primary emphasis of the selected studies cantered on medical students, the deliberate incorporation of nursing and allied health professionals emphasises a progressing recognition of the significance of fostering interprofessionality across diverse healthcare disciplines. This collaborative approach not only allows for the realistic application of NTS but also facilitates the cultivation of teamwork and collaborative leadership skills within the simulation context [36, 42, 54, 60, 62, 64, 68, 70, 73]. This emerging trend highlights the necessity for future training programs to conscientiously account for the distinctive NTS requirements inherent to diverse healthcare professionals, thereby fostering a more comprehensive and effective approach to medical simulations.

These findings summarise how NTS education is currently being delivered through simulation and highlight effective strategies that can be used to develop consistent, effective and contextually appropriate learning. The insights described above can be used as practical guidance for educators aiming to embed NTS training via simulation into their respective curricula.

### Limitations

When conducting this scoping review, only articles in English were included. A more extensive search from other languages would be beneficial to foster inclusivity and diversity. Furthermore, despite a broad predetermined search strategy, potential studies could have been overlooked in the absence of other search terms and an omission of grey literature. The process of a scoping review on title and abstract screening prior to full-text review introduces efficiency at the cost of potentially excluding suitable papers. Additionally, within this article, we have mainly focused on physical simulations rather than virtual environments, which have been discussed in other literature [122, 123]. As the target population for this review is undergraduate medical students, the results may be limited in their generalisability to other populations such as students from other disciplines or post-graduate healthcare professionals. Therefore, future studies may explore this difference and investigate how training non-technical skills may differ across diverse healthcare disciplines or professional experience. Furthermore, some studies have adapted existing assessment tools to better suit group dynamics and educational contexts. Hence, to better understand the impact of modified tools, future research could potentially investigate how well these adapted tools perform across different educational settings and simulation contexts, particularly in terms of reliability, validity, and usability.

## Conclusions

Our review has identified key components of NTS training, summarised into themes including pre-simulation preparation, thoughtful simulation design, post-simulation reinforcement, assessment and optimal learning environment. The strategies within each component offer insights for enhancing the quality, consistency and impact of simulation-based NTS education. By collating these practices, this review offers educators clear, evidence-based guidance for successful design and delivery of NTS training in undergraduate medical education.

## Supplementary Information

Supplementary Material 1: Supplementary Table 1. Pre-simulation activities conducted. Supplementary Table 2. Simulation activities. Supplementary Table 3. Structure of post-simulation debriefing and feedback. Supplementary Table 4. Overview of Non-Technical Skills implemented. Supplementary Table 5. Observational Assessment Tools Employed for Evaluating Non-Technical Skills Across the Reviewed Studies. Supplementary Table 6. Studies exploring learning environment and inter professional education.

## Data Availability

No datasets were generated or analysed during the current study.

## Appendix 1

### Search strategy

**Table:** Search Terms: (‘Non-Technical Skills’ OR ‘Human Factors’ OR ‘Human Ergonomics’ OR ‘Human Behaviour’ OR ‘Behavioural Skills’) AND (Medical OR Clinical OR Surgical) AND (Simulation) AND (‘Undergraduate Medical Education’ OR ‘Medical Student’ OR ‘Undergraduate Doctor’)

**PUBMED **(*from inception to 17 June 2025*)		
**No.**	**Search Details**	**Results**
**1**	‘Undergraduate Medical Education’[Title/Abstract] OR ‘medical student*’[Title/Abstract] OR ‘undergraduate doctor*’[Title/Abstract] OR ‘student doctor*’[Title/Abstract] OR ‘trainee doctor*’[Title/Abstract]	64,033
**2**	‘medic’[All Fields] OR ‘medical’[All Fields] OR ‘medicalization’[MeSH Terms] OR ‘medicalization’[All Fields] OR ‘medicalizations’[All Fields] OR ‘medicalize’[All Fields] OR ‘medicalized’[All Fields] OR ‘medicalizes’[All Fields] OR ‘medicalizing’[All Fields] OR ‘medically’[All Fields] OR ‘medicals’[All Fields] OR ‘medicated’[All Fields] OR ‘medication s’[All Fields] OR ‘medics’[All Fields] OR ‘pharmaceutical preparations’[MeSH Terms] OR (‘pharmaceutical’[All Fields] AND ‘preparations’[All Fields]) OR ‘pharmaceutical preparations’[All Fields] OR ‘medication’[All Fields] OR ‘medications’[All Fields] OR (‘ambulatory care facilities’[MeSH Terms] OR (‘ambulatory’[All Fields] AND ‘care’[All Fields] AND ‘facilities’[All Fields]) OR ‘ambulatory care facilities’[All Fields] OR ‘clinic’[All Fields] OR ‘clinic s’[All Fields] OR ‘clinical’[All Fields] OR ‘clinically’[All Fields] OR ‘clinicals’[All Fields] OR ‘clinics’[All Fields]) OR (‘surgical procedures, operative’[MeSH Terms] OR (‘surgical’[All Fields] AND ‘procedures’[All Fields] AND ‘operative’[All Fields]) OR ‘operative surgical procedures’[All Fields] OR ‘surgical’[All Fields] OR ‘surgically’[All Fields] OR ‘surgicals’[All Fields])	16,047,334
**3**	‘non technical skill*’[All Fields] OR ‘human factor*’[All Fields] OR ‘human ergonomic*’[All Fields] OR ‘human behaviour*’[All Fields] OR ‘Behavioural skill*’[All Fields}	22,617
**4**	‘simulation*’[All Fields]	754,156
**5**	#1 AND #2 AND #3 AND #4	116
**SCOPUS ***(from inception to 17 June 2025)*		
**No.**	**Search Details**	**Results**
1	TITLE-ABS-KEY (‘Undergraduate Medical Education’ OR ‘Medical Student*’ OR ‘Undergraduate Doctor*’ OR ‘ Student Doctor*’ OR ‘Trainee Doctor*’)	120,126
2	ALL (medical OR clinical OR surgical)	32,068,886
3	TITLE-ABS-KEY (‘Non-Technical Skill*’ OR ‘Human Factor*’ OR ‘Human Ergonomic*’ OR ‘Human Behaviour*’ OR ‘Behavioural skill*’)	75,886
4	TITLE-ABS-KEY (simulation*)	5,358,675
**5**	#1 AND #2 AND #3 AND #4	166
**WEB OF SCIENCE ***(from inception to 17 June 2025)*		
**No.**	**Search Query**	**Results**
1	TS=(‘Undergraduate Medical Education’ OR ‘Medical Student*’ OR ‘Undergraduate Doctor*’ OR ‘Student Doctor*’ OR ‘Trainee Doctor*’)	74,337
2	ALL=(medical OR clinical OR surgical)	https://www-webofscience-com.ezproxy-prd.bodleian.ox.ac.uk/wos/woscc/summary/47a17dd0-586f-4239-9e1a-c962c3531efc-01691aa4ec/relevance/124,188,407
3	TS=(‘Non-Technical Skill*’ OR ‘Human Factor*’ OR ‘Human Ergonomic*’ OR ‘Human Behaviour*’ OR ‘Behavioural Skill*’)	https://www-webofscience-com.ezproxy-prd.bodleian.ox.ac.uk/wos/woscc/summary/6f0bd2d3-c3cb-45ff-a248-96d9c25aac3c-01691aa577/relevance/143,583
4	TS=(simulation*)	https://www-webofscience-com.ezproxy-prd.bodleian.ox.ac.uk/wos/woscc/summary/335b2b5d-945a-4a85-8cc4-44c8c25c4b44-01691aa5d3/relevance/13,851,747
5	#1 AND #2 AND #3 AND #4	https://www-webofscience-com.ezproxy-prd.bodleian.ox.ac.uk/wos/woscc/summary/3216e2bc-5b72-44b9-add2-b12e319c5349-01691aa668/relevance/1124

## Appendix 2

### Inclusion and exclusion criteria

**Table:**

**Inclusion criteria**	**Exclusion criteria (Any may apply)**
**Focus of study**	
Studies that described the delivery of non-technical or behavioural skills training in undergraduate medical simulations.	Technical skills training Postgraduate medical education
**Characteristics of the intervention**	
Any method of delivering NTS or behavioural skills training, including and not limited to pre-simulation briefing, pre-simulation learning activities, type of simulation, or debriefing.	Articles that focus on the development of theoretical frameworks, curricula, or tools without detailing their application in a simulation setting. Articles that discuss learners’ experiences and perceptions without providing details of the simulation.
**Population**	
Undergraduate medical students	Graduate doctors Specialty trainees Studies involving health professionals without the inclusion of undergraduate medical students.
**Study characteristics**	
Primary research articles Qualitative and/or quantitative methods	Review articles Editorials and opinion pieces
**Publication characteristics**	
Full text articles English language only From inception until 17 June 2025	No full text available Studies not in English

## Appendix 3

### Quality assessment

JBI critical appraisal checklist for analytical cross sectional studies

Items:

**Table:**

1. Were the criteria for inclusion in the sample clearly defined?
2. Were the study subjects and the setting described in detail?
3. Was the exposure measured in a valid and reliable way?
4. Were objective, standard criteria used for measurement of the condition?
5. Were confounding factors identified?
6. Were strategies to deal with confounding factors stated?
7. Were the outcomes measured in a valid and reliable way?
8. Was appropriate statistical analysis used?

**Table:**

Year	Author	1	2	3	4	5	6	7	8
2023	Martinho	n	y	y	y	y	y	y	y
2021	Flentje	y	y	y	y	y	y	y	y
2018	MacMillan	y	y	y	y	y	y	y	y

*y* yes, *n* no

JBI critical appraisal checklist for cohort studies

Items:

**Table:**

1. Were the two groups similar and recruited from the same population?
2. Were the exposures measured similarly to assign people to both exposed and unexposed groups?
3. Was the exposure measured in a valid and reliable way?
4. Were confounding factors identified?
5. Were strategies to deal with confounding factors stated?
6. Were the groups/participants free of the outcome at the start of the study (or at the moment of exposure)?
7. Were the outcomes measured in a valid and reliable way?
8. Was the follow up time reported and sufficient to be long enough for outcomes to occur?
9. Was follow up complete, and if not, were the reasons to loss to follow up described and explored?
10. Were strategies to address incomplete follow up utilized?
11. Was appropriate statistical analysis used?

**Table:**

Year	Author	1	2	3	4	5	6	7	8	9	10	11
2023	Moll-Khosrawi	y	y	y	y	y	y	y	y	n	y	y
2021	Łoś	y	y	y	y	y	y	y	y	y	y	y
2021	Nicolaides	y	y	y	y	y	y	y	y	y	y	y
2020	Nicolaides	y	y	y	y	y	y	y	y	y	y	y
2018	Mannella	y	y	y	y	y	y	y	y	y	y	y
2018	Parker	y	y	y	y	y	y	y	y	y	y	y
2010	Cahan	y	y	y	y	y	y	y	y	y	y	y

*y* yes, *n* no

JBI checklist for quasi-experimental studies

Items:

**Table:**

1. Is it clear in the study what is the “cause” and what is the “effect” (i.e. there is no confusion about which variable comes first)?
2. Was there a control group?
3. Were participants included in any comparisons similar?
4. Were the participants included in any comparisons receiving similar **intervention**, other than the exposure or intervention of interest?
5. Were there multiple measurements of the outcome, both pre and post the intervention/exposure?
6. Were the outcomes of participants included in any comparisons measured in the same way?
7. Were outcomes measured in a reliable way?
8. Was follow-up complete and if not, were differences between groups in terms of their follow-up adequately described and analyzed?
9. Was appropriate statistical analysis used?

**Table:**

Year	Author	1	2	3	4	5	6	7	8	9
2022	Lee	y	n	y	y	y	y	y	u	y
2022	Major	y	n	y	y	y	y	y	u	y
2021	Boukatta	y	n	y	y	y	y	y	y	y
2021	Taylor	y	n	y	y	y	y	y	n, y	y
2020	Seale	y	n	y	y	n	y	u	u	n
2019	Backhouse	y	n	y	y	y	y	y	y	u
2019	Cha	y	n	y	y	y	y	y	y	y
2019	Seale	y	n	y	y	y	y	y	y	y
2019	Young	y	n	y	y	y	y	y	y	y
2018	Nagraj	y	n	y	y	y	y	y	y	y
2018	Sideris	y	n	y	y	y	y	y	y	y
2016	Jorm	y	n	y	y	y	y	y	n, y	y
2016	Levinson	y	n	y	y	y	y	y	n, y	y
2016	Partecke	y	n	y	y	y	y	y	u	y
2016	Reime	y	n	y	y	y	y	y	n, u	y
2015	Harvey	y	n	y	y	y	y	y	n, u	y
2012	Shelton	y	n	y	y	y	y	y	u	y

*y* yes, *n* no, *u* unclear

JBI critical appraisal checklist for qualitative research

Items:

1. Is there congruity between the stated philosophical perspective and the research methodology?
2. Is there congruity between the research methodology and the research question or objectives?
3. Is there congruity between the research methodology and the methods used to collect data?
4. Is there congruity between the research methodology and the representation and analysis of data?
5. Is there congruity between the research methodology and the interpretation of results?
6. Is there a statement locating the researcher culturally or theoretically?
7. Is the influence of the researcher on the research, and vice- versa, addressed?
8. Are participants, and their voices, adequately represented?
9. Is the research ethical according to current criteria or, for recent studies, and is there evidence of ethical approval by an appropriate body?
10. Do the conclusions drawn in the research report flow from the analysis, or interpretation, of the data?

**Table:**

Year	Author	1	2	3	4	5	6	7	8	9	10
2022	Pollard	y	y	y	y	y	n	y	y	y	y
2021	Tervajärvi	y	y	y	y	y	y	y	y	y	y
2021	Yates	y	y	y	y	y	y	y	y	y	y
2020	Carter	y	y	y	y	y	y	y	y	y	y
2020	Kerins	y	y	y	y	y	y	y	y	y	y
2020	Pal	y	y	y	y	y	y	y	y	y	y
2018	Jakobsen	y	y	y	y	y	y	y	y	y	y
2015	Gregory	y	y	y	y	y	y	y	y	y	y
2015	Thomas	y	y	y	y	y	y	y	y	y	y
2014	Dickinson	y	y	y	y	y	y	y	y	y	y

*y* yes, *n* no

JBI critical appraisal tool for assessment of risk of bias for randomized controlled trials

Items:

1. Was true randomization used for assignment of participants to intervention groups?
2. Was allocation to intervention groups concealed?
3. Were intervention groups similar at the baseline?
4. Were participants blind to intervention assignment?
5. Were those delivering the intervention blind to intervention assignment?
6. Were intervention groups treated identically other than the intervention of interest?
7. Were outcome assessors blind to intervention/assignment?
8. Were outcomes measured in the same way for intervention groups?
9. Were outcomes measured in a reliable way
10. Was follow up complete and if not, were differences between groups in terms of their follow up adequately described and analysed?
11. Were participants analysed in the groups to which they were randomized?
12. Was appropriate statistical analysis used?
13. Was the trial design appropriate and any deviations from the standard RCT design (individual randomization, parallel groups) accounted for in the conduct and analysis of the trial

**Table:**

Year	Author	1	2	3	4	5	6	7	8	9	10	11	12	13
2023	Sá-Couto	y	y	y	y	y	y	y	y	y	y	y	y	y
2022	Nabecker	y	y	y	y	n	y	n	y	y	y	y	y	y
2021	Freytag	y	y	n	y	u	y	u	y	y	y	y	y	y
2021	Moll-Khosrawi	y	y	y	y	n	y	u	y	y	y	y	y	y
2021	Wai	y	y	y	u	u	y	u	y	y	y	y	y	y
2019	Eismann	y	y	y	y	n	y	N/A	y	y	y	y	y	y
2017	Fukuta	y	y	y	y	n	y	u	y	y	y	y	y	y
2017	Hagemann	y	y	y	y	u	y	y	y	y	y	y	y	y
2016	Morrissey	y	y	y	y	u	y	N/A	y	y	y	y	y	y
2015	Cortegiani	y	y	y	y	u	y	y	y	y	y	y	y	y
2024	Botelho	y	y	y	y	n	y	y	y	y	y	y	y	y
2024	Ohlenburg	y	y	y	y	n	y	y	y	y	y	y	y	y
2024	Jaffrelot	y	y	y	y	n	y	y	y	y	y	y	y	y
2024	Whallett	y	y	y	y	n	y	y	y	y	y	y	y	y

*y* yes, *n* no, *u* unclear, *N*/*A* not applicable

## Abbreviations

AI: Artificial intelligence
NTS: Non-technical skills
VR: Virtual reality
