# Supplementary material for: Integrating non-technical skills into undergraduate medical simulation: a scoping review and thematic analysis of current practices
Source: Adv Simul (Lond). 2025 Oct 21;10:49. doi: 10.1186/s41077-025-00377-9 (PMC12542256; doi:10.1186/s41077-025-00377-9)
Supplement: Supplementary file 1 — Supplementary Material 1: Supplementary Table 1. Pre-simulation activities conducted. Supplementary Table 2. Simulation activities. Supplementary Table 3. Structure of post-simulation debriefing and feedback. Supplementary Table 4. Overview of Non-Technical Skills implemented. Supplementary Table 5. Observational Assessment Tools Employed for Evaluating Non-Technical Skills Across the Reviewed Studies. Supplementary Table 6. Studies exploring learning environment and inter professional education. [file 41077_2025_377_MOESM1_ESM.docx]

**Supplementary Tables**

Supplementary Table 1: Pre-simulation activities conducted.

| **Pre-Simulation Activity** | **Technical Skills (TS)** | **Non-Technical Skills (NTS)** | **Studies** |
| --- | --- | --- | --- |
| **Lectures** | ✓ | ✓ | [1-4] |
|  | ✓ |  | [5-8] |
|  |  | ✓ | [9, 10] |
| **E-Learning** | ✓ | ✓ | [7, 11, 12] |
|  |  | ✓ | [13] |
| **Videos** | ✓ | ✓ | [14, 15] |
|  |  | ✓ | [16] |
| **Collaborative learning** | ✓ | ✓ | [11, 17] |

Supplementary Table 2: Simulation activities.

| **Type of Simulation** | **Studies** |
| --- | --- |
| Individual Simulation Scenarios | [1-9, 11-41] |
| Simulated Wards | [26, 42-47] |
| Virtual Reality Simulation | [10] |
| Recorded Video | [16] |
| Gamification within an Escape Room | [48] |
| Large-Class Simulation Involving a Natural Disaster | [49] |
| Simulated Queries through Various Interactions | [50] |
| Unspecified | [51] |

Supplementary Table 3: Structure of post-simulation debriefing and feedback.

| **Feedback/Debriefing Method** | **Study** |
| --- | --- |
| Individual Teamwork Observation and Feedback Tool (iTOFT) | [31, 46] |
| TeamGAINS | [23, 39] |
| Gather-Analyse-Summarise (GAS) | [29] |
| Debrief Diamond | [24] |
| Behavioural marker system | [26] |
| Promoting Excellence and Reflective Learning in Simulation (PEARLS) | [36] |
| Saw, Else, Thinks, Goals, Offers (SET-GO) | [44] |
| Gibbs cycle reflection | [42] |
| Recorded video footage | [5, 11, 20, 35, 39] |
| After-action review | [48] |
| Near peers | [25] |
| Unspecified | [3, 6-8, 12, 15-19, 21, 24, 28, 32-34, 38, 40, 43, 47, 49] |

Supplementary Table 4: Overview of Non-Technical Skills (NTS) implemented.

| **Year** | **Study** | **Location** | **Teamwork** | **Prioritisation** | **Situational awareness** | **Leadership** | **Decision Making** | **Safety behaviours** | **Patient communication** | **Wellbeing** |
| --- | --- | --- | --- | --- | --- | --- | --- | --- | --- | --- |
| 2024 | Botelho | Canada, Brazil | ✓ |  | ✓ | ✓ | ✓ |  |  |  |
| 2024 | Ohlenburg | Germany | ✓ | ✓ |  | ✓ |  |  |  |  |
| 2024 | Jaffrelot | France | ✓ |  | ✓ | ✓ | ✓ |  |  |  |
| 2024 | Whallett | United Kingdom | ✓ | ✓ | ✓ |  | ✓ | ✓ |  |  |
| 2023 | Martinho | Portugal | ✓ |  | ✓ | ✓ | ✓ |  |  | ✓ |
| 2023 | Moll-Khosrawi | Germany | ✓ | ✓ |  | ✓ | ✓ |  |  |  |
| 2023 | Sá-Couto | Portugal | ✓ | ✓ |  | ✓ |  |  |  |  |
| 2022 | Lee | South Korea | ✓ | ✓ |  | ✓ |  |  |  |  |
| 2022 | Major | Germany, Italy, Belgium, Netherlands, Romania, Portugal, Syria | ✓ |  |  |  | ✓ |  |  | ✓ |
| 2022 | Nabecker | Switzerland | ✓ |  | ✓ | ✓ |  |  |  |  |
| 2022 | Pollard | United Kingdom | ✓ | ✓ |  |  | ✓ |  | ✓ |  |
| 2021 | Boukatta | Morocco | ✓ | ✓ |  | ✓ |  |  |  |  |
| 2021 | Flentje | Germany | ✓ |  |  |  |  |  |  |  |
| 2021 | Freytag | Berlin | ✓ | ✓ | ✓ | ✓ | ✓ | ✓ |  |  |
| 2021 | Łoś | Poland | ✓ |  |  |  |  |  |  |  |
| 2021 | Moll-Khosrawi | Germany | ✓ | ✓ |  | ✓ | ✓ |  |  |  |
| 2021 | Nicolaides | European Union | ✓ |  |  |  |  |  |  |  |
| 2021 | Taylor | Dominican Republic | ✓ | ✓ | ✓ | ✓ | ✓ |  |  | ✓ |
| 2021 | Tervajärvi | Finland | ✓ |  |  |  |  |  | ✓ |  |
| 2021 | Wai | Hong Kong | ✓ | ✓ | ✓ | ✓ | ✓ |  |  |  |
| 2021 | Yates | Australia | ✓ |  |  |  |  |  | ✓ | ✓ |
| 2020 | Carter | United Kingdom | ✓ |  | ✓ |  |  | ✓ | ✓ |  |
| 2020 | Kerins | United Kingdom | ✓ | ✓ | ✓ |  | ✓ | ✓ |  |  |
| 2020 | Nicolaides | Europe | ✓ | ✓ |  | ✓ |  |  |  |  |
| 2020 | Pal | United Kingdom | ✓ | ✓ | ✓ | ✓ |  | ✓ | ✓ |  |
| 2020 | Seale | United Kingdom | ✓ |  | ✓ |  | ✓ | ✓ |  |  |
| 2019 | Backhouse | United Kingdom | ✓ |  |  |  |  |  |  |  |
| 2019 | Cha | United States | ✓ |  | ✓ | ✓ | ✓ | ✓ | ✓ |  |
| 2019 | Eismann | Germany | ✓ | ✓ | ✓ |  | ✓ |  |  |  |
| 2019 | Seale | United Kingdom | ✓ |  | ✓ |  |  |  |  |  |
| 2019 | Young | United Kingdom | ✓ | ✓ | ✓ |  |  |  |  |  |
| 2018 | Jakobsen | Norway | ✓ |  | ✓ | ✓ |  |  |  | ✓ |
| 2018 | MacMillan | United Kingdom | ✓ | ✓ | ✓ |  | ✓ | ✓ |  |  |
| 2018 | Mannella | Italy |  |  |  |  | ✓ |  |  |  |
| 2018 | Nagraj | United Kingdom | ✓ |  |  |  |  |  |  |  |
| 2018 | Parker | United Kingdom | ✓ | ✓ |  |  |  |  |  |  |
| 2018 | Sideris | Greece | ✓ |  |  | ✓ |  |  |  |  |
| 2017 | Fukuta | United Kingdom | ✓ | ✓ | ✓ | ✓ | ✓ |  |  |  |
| 2017 | Hagemann | Germany | ✓ | ✓ | ✓ |  | ✓ |  |  |  |
| 2016 | Jorm | Australia | ✓ | ✓ |  | ✓ | ✓ |  |  |  |
| 2016 | Levinson | Not mentioned | ✓ | ✓ |  | ✓ | ✓ |  | ✓ |  |
| 2016 | Morrissey | United Kingdom | ✓ |  |  | ✓ |  |  |  |  |
| 2016 | Partecke | Germany | ✓ |  |  |  |  |  |  |  |
| 2016 | Reime | Norway | ✓ |  | ✓ |  |  |  |  |  |
| 2015 | Cortegiani | Italy | ✓ |  |  | ✓ | ✓ |  |  |  |
| 2015 | Gregory | United Kingdom |  |  | ✓ |  |  |  |  |  |
| 2015 | Harvey | United Kingdom | ✓ | ✓ | ✓ | ✓ | ✓ |  | ✓ | ✓ |
| 2015 | Thomas | United Kingdom | ✓ | ✓ | ✓ |  |  | ✓ |  |  |
| 2014 | Dickinson | United Kingdom | ✓ | ✓ | ✓ | ✓ | ✓ |  |  |  |
| 2012 | Shelton | United Kingdom |  |  | ✓ |  |  |  |  |  |
| 2010 | Cahan | United States | ✓ | ✓ |  |  |  | ✓ | ✓ |  |

Supplementary Table 5: Observational Assessment Tools Employed for Evaluating Non-Technical Skills (NTS) Across the Reviewed Studies

| **Observational Assessment Tool** | **Study** |
| --- | --- |
| Teamwork Emergency Assessment Measure (TEAM) | [8, 15, 28, 35] |
| Anaesthesia Non-Technical Skills (ANTS) | [9, 23] |
| Anaesthesiology students’ Non-Technical Skills (AS-NTS) | [3, 6] |
| Individual Teamwork Observation and Feedback Tool (iTOFT) | [31, 46] |
| Medical Students' Non-Technical Skills (Medi-StuNTS) | [26, 41] |
| Modified NOTECHS | [22, 32] |
| Observational form for peer evaluation | [20, 38] |
| Ottawa Crisis Resource Management (CRM) score | [14, 30] |
| Human Factors Attitude Survey (HFAS), Ottawa Global Rating Scale, Team-Based Learning Student Assessment Instrument (TBL-SAI) | [11] |
| Trauma Team Performance Observation Tool (TPOT) and  Team STEPPS Teamwork Attitudes Questionnaire (T-TAQ) | [2] |
| A–E non-technical skills guidance sheet | [27] |
| 5-point scale derived from vignettes composed by participants | [16] |
| Evaluation of collective orientation | [39] |
| N-PIPS questionnaire developed by the Royal Cornwall Hospital | [25] |
| Non-technical skill evaluation scale (NTS scale) - unvalidated | [1] |
| Situation Present Assessment Method for iOS (iSPAM) | [50] |
| Video-reflexive Ethnography (VRE) techniques combined with interviews | [13] |
| Visual Analog Scale | [37] |
| Ottawa Global Rating Scale (OGRS) | [40] |
| Non-Technical Skills for Surgeons (NOTSS) | [12] |

Supplementary Table 6: Studies exploring learning environment and interprofessional education.

| **Label** | **Studies** |
| --- | --- |
| Learning Environment | [5, 6, 8, 19, 23, 26, 30, 32, 34, 36, 38, 43, 44, 49] |
| Interprofessional Learning | [4, 5, 11, 20, 21, 24, 25, 32, 33, 36, 39, 46] |

1. Mannella P, Antonelli R, Montt-Guevara MM, Caretto M, Palla G, Giannini A *et al*. Simulation of childbirth improves clinical management capacity and self-confidence in medical students. BMJ Simul Technol Enhanc Learn. 2018;4:184-189.

2. Martinho B, Ferreira L, Koch MJ, Madeira F, Santos E, Baptista S *et al*. Observational Study About the Impact of Simulation Training of Non-Technical Skills on Teamwork: Towards a Paradigm Shift in Undergraduate Medical Training. Acta Med Port. 2023.

3. Moll-Khosrawi P, Zöllner C, Cencin N, Schulte-Uentrop L. Flipped learning enhances non-technical skill performance in simulation-based education: a randomised controlled trial. BMC Med Educ. 2021;21:353.

4. Partecke M, Balzer C, Finkenzeller I, Reppenhagen C, Hess U, Hahnenkamp K *et al*. Interprofessional Learning - Development and Implementation of Joint Medical Emergency Team Trainings for Medical and Nursing Students at Universitätsmedizin Greifswald. GMS J Med Educ. 2016;33:Doc32.

5. Jakobsen RB, Gran SF, Grimsmo B, Arntzen K, Fosse E, Frich JC *et al*. Examining participant perceptions of an interprofessional simulation-based trauma team training for medical and nursing students. Journal of Interprofessional Care. 2018;32:80-88.

6. Moll-Khosrawi P, Küllmei J, Zöllner C, Schulte-Uentrop L. Efficacy of an Integrated Simulation-Based Education Approach to Train Non-Technical Skills in Medical Undergraduate Students. Education Sciences. 2023;13.

7. Morrissey B, Jacob H, Harnik E, Mackay K, Moreiras J. Simulation in undergraduate paediatrics: a cluster-randomised trial. Clin Teach. 2016;13:337-342.

8. Nicolaides M, Theodorou E, Emin EI, Theodoulou I, Andersen N, Lymperopoulos N *et al*. Team performance training for medical students: Low vs high fidelity simulation. Ann Med Surg (Lond). 2020;55:308-315.

9. Hagemann V, Herbstreit F, Kehren C, Chittamadathil J, Wolfertz S, Dirkmann D *et al*. Does teaching non-technical skills to medical students improve those skills and simulated patient outcome? Int J Med Educ. 2017;8:101-113.

10. Pal S, Benson R, Duvall P, Taylor-Jones V. Do innovative immersive virtual reality simulation videos have a role to play in teaching non-technical skills and increasing preparedness for clinical placements for medical students? MedEdPublish. 2020;9:164.

11. Wai AKC, Lam VSF, Ng ZLH, Pang MTH, Tsang VWY, Lee JJJ *et al*. Exploring the role of simulation to foster interprofessional teamwork among medical and nursing students: A mixed-method pilot investigation in Hong Kong. Journal of Interprofessional Care. 2021;35:890-898.

12. Botelho F, Gerk A, Harley JM, Poenaru D. Improving Pediatric Trauma Education by Teaching Non-technical Skills: A Randomized Controlled Trial. Journal of Pediatric Surgery. 2024;59:874-888.

13. Carter H, Hanks S, Gale T. A qualitative study using hybrid simulation to explore the impacts of human factors e-learning on behaviour change. Adv Simul (Lond). 2020;5:20.

14. Fukuta J, Morgan J. First-person perspective video to enhance simulation. Clin Teach. 2018;15:231-235.

15. Ohlenburg H, Arnemann P-H, Hessler M, Görlich D, Zarbock A, Friederichs H. Flipped Classroom: Improved team performance during resuscitation training through interactive pre-course content – a cluster-randomised controlled study. BMC Medical Education. 2024;24.

16. Cahan MA, Larkin AC, Starr S, Wellman S, Haley HL, Sullivan K *et al*. A human factors curriculum for surgical clerkship students. Arch Surg. 2010;145:1151-1157.

17. Levinson M, Kelly D, Zahariou K, Johnson M, Jackman C, Mackenzie S. Description and student self-evaluation of a pilot integrated small group learning and simulation programme for medical students in the first clinical year. Intern Med J. 2017;47:211-216.

18. Dickinson M, Pimblett M, Hanson J, Davis M. Reflecting reality: pager simulations in undergraduate education. Clin Teach. 2014;11:421-424.

19. Cortegiani A, Russotto V, Montalto F, Iozzo P, Palmeri C, Raineri SM *et al*. Effect of High-Fidelity Simulation on Medical Students' Knowledge about Advanced Life Support: A Randomized Study. PLoS One. 2015;10:e0125685.

20. Reime MH, Johnsgaard T, Kvam FI, Aarflot M, Breivik M, Engeberg JM *et al*. Simulated settings; powerful arenas for learning patient safety practices and facilitating transference to clinical practice. A mixed method study. Nurse Educ Pract. 2016;21:75-82.

21. Nagraj S, Harrison J, Hill L, Bowker L, Lindqvist S. Promoting collaboration in emergency medicine. Clin Teach. 2018;15:500-505.

22. Cha JS, Anton NE, Mizota T, Hennings JM, Rendina MA, Stanton-Maxey K *et al*. Use of non-technical skills can predict medical student performance in acute care simulated scenarios. Am J Surg. 2019;217:323-328.

23. Eismann H, Palmaers T, Tsvetanov S, Hagemann V, Flentje M. Changes of collective orientation through a medical student's anaesthesia simulation course - simulation-based training study with non-technical skills debriefing versus medical debriefing. BMC Med Educ. 2019;19:337.

24. Seale J, Ikram S, Whittingham L, Butchers C. Combining medical, physiotherapy and nursing undergraduates in high-fidelity simulation: determining students' perceptions. BMJ Simul Technol Enhanc Learn. 2019;5:108-110.

25. Young M, Wilkinson T. Near-peer interprofessional simulation training in an undergraduate setting. BMJ Simulation & Technology Enhanced Learning. 2019;5:111-113.

26. Kerins J, Smith SE, Phillips EC, Clarke B, Hamilton AL, Tallentire VR. Exploring transformative learning when developing medical students' non‐technical skills. Medical Education. 2020;54:264-274.

27. Seale J, Khan A, Hirons B, Butchers C. ABCDE: Directing Student Observation During High-Fidelity Simulation. Med Sci Educ. 2020;30:1347-1349.

28. Boukatta B, El Bouazzaoui A, Touzani S, Houari N, Benmaamar S, Kanjaa N. A Case Study On Significance Of Medical Simulation For Soft Skills Training In Emergency Medical Crisis Management. Journal of Medical and Surgical Research. 2021;8:990-996.

29. Freytag J, Stroben F, Hautz WE, Penders D, Kämmer JE. Effects of using a cognitive aid on content and feasibility of debriefings of simulated emergencies. GMS J Med Educ. 2021;38:Doc95.

30. Łoś K, Chmielewski J, Cebula G, Bielecki T, Torres K, Łuczyński W. Relationship between mindfulness, stress, and performance in medical students in pediatric emergency simulations. GMS J Med Educ. 2021;38:Doc78.

31. Nicolaides M, Theodorou E, Hanrahan JG, Theodoulou I, Emin EI, Papalois A *et al*. Advancing Medical Students' Non-technical Skills in a Group-Based Setting. J Invest Surg. 2021;34:39-43.

32. Taylor S, Avrith N, Loo G, Millán R, Wyler BA, McVane B *et al*. Impact of a focused trauma course on retention of provider skills, knowledge and confidence at a regional hospital in the Dominican Republic. Injury. 2021;52:2526-2533.

33. Tervajärvi L, Hutri-Kähönen N, Rautiola AM. Student-LED interprofessional sequential simulation improves communication and teamwork. Nurse Educ Pract. 2021;51:102983.

34. Yates N, Purdy E, Braganza S, Alsaba N, Spooner A, Smith J *et al*. Can simulation foster resilience in medical students? BMJ Simul Technol Enhanc Learn. 2021;7:50-51.

35. Lee J, Lee JH. Effects of simulation-based education for neonatal resuscitation on medical students' technical and non-technical skills. PLoS One. 2022;17:e0278575.

36. Major S, Krage R, Lazarovici M. SimUniversity at a distance: a descriptive account of a team-based remote simulation competition for health professions students. Adv Simul (Lond). 2022;7:6.

37. Nabecker S, Huwendiek S, Seidl C, Hana A, Theiler L, Greif R. Assessment of Human Factors After Advanced Life Support Courses Comparing Simulated Team and Real Team Assessment: A Randomized Controlled Cohort Trial. Front Cardiovasc Med. 2022;9:840114.

38. Sá-Couto C, Rodrigues D, Gouveia M. Debriefing or Feedback: Exploring the Impact of Two Post-Scenario Discussion Methods in the Acquisition and Retention of Non-Technical Skills. Acta Med Port. 2023;36:34-41.

39. Flentje M, Hagemann V, Breuer G, Bintaro P, Eismann H. Change of collective orientation through an interprofessional training with medical students and student nurses depending on presence and professional group. BMC Med Educ. 2021;21:365.

40. Jaffrelot M, Boet S, Floch Y, Garg N, Dubois D, Laparra V *et al*. Learning with our peers: peer-led versus instructor-led debriefing for simulated crises, a randomized controlled trial. Korean Journal of Anesthesiology. 2024;77:265-272.

41. Whallett M, Mahesh S, Whittaker J, Crichton A, Ahmed U. Evaluating the Impact of Virtual Reality on Medical Students’ Skills Performance in High- Fidelity Simulation: A Randomised Controlled Trial Pilot. Education for Health. 2024;37:367-376.

42. Gregory A, Hogg G, Ker J. Innovative teaching in situational awareness. Clin Teach. 2015;12:331-335.

43. Harvey R, Mellanby E, Dearden E, Medjoub K, Edgar S. Developing non-technical ward-round skills. Clin Teach. 2015;12:336-340.

44. Thomas I, Nicol L, Regan L, Cleland J, Maliepaard D, Clark L *et al*. Driven to distraction: a prospective controlled study of a simulated ward round experience to improve patient safety teaching for medical students. BMJ Qual Saf. 2015;24:154-161.

45. MacMillan C, Hogg G. Exploring student perceptions of ward simulation as an exercise to improve decision-making skills in the clinical context. MedEdPublish. 2018;7:18.

46. Parker R, Hodierne L, Anderson ES, Davies RS, Elloy M. Academic ability and teamworking in medical students. Clin Teach. 2019;16:209-213.

47. Pollard J, Tombs M. Teaching Undergraduate Medical Students Non-Technical Skills: An Evaluation Study of a Simulated Ward Experience. Adv Med Educ Pract. 2022;13:485-494.

48. Backhouse A, Malik M. Escape into patient safety: bringing human factors to life for medical students. BMJ Open Qual. 2019;8:e000548.

49. Jorm C, Roberts C, Lim R, Roper J, Skinner C, Robertson J *et al*. A large-scale mass casualty simulation to develop the non-technical skills medical students require for collaborative teamwork. BMC Med Educ. 2016;16:83.

50. Shelton CL, Kinston R, Molyneux AJ, Ambrose LJ. Real-time situation awareness assessment in critical illness management: adapting the situation present assessment method to clinical simulation. BMJ Qual Saf. 2013;22:163-167.

51. Sideris M, Hanrahan J, Tsoulfas G, Theodoulou I, Dhaif F, Papalois V *et al*. Developing a novel international undergraduate surgical masterclass during a financial crisis: our 4-year experience. Postgrad Med J. 2018;94:263-269.
